# A paired comparison of nerve dimensions using B-Mode ultrasound and shear wave elastography during regional anaesthesia

**DOI:** 10.1177/1742271X221091726

**Published:** 2022-05-09

**Authors:** Heather Lockwood, Graeme A McLeod

**Affiliations:** 1 School of Medicine, University of Dundee, Dundee, UK; 2 Institute of Academic Anesthesia, School of Medicine, University of Dundee, Dundee, UK

**Keywords:** Clinical–physics and engineering, quality assurance–physics and engineering, artificial intelligence, statistics, anaesthesia

## Abstract

**Introduction:**

Shear wave elastography (SWE) presents nerves in colour, but the dimensions of its colour maps have not been validated with paired B-Mode nerve images. Our primary objective was to define the bias and limits of agreement of SWE with B-Mode nerve diameter. Our secondary objectives were to compare nerve area and shape, and provide a clinical standard for future application of new colour imaging technologies such as artificial intelligence.

**Materials and Methods:**

Eleven combined ultrasound-guided regional nerve blocks were conducted using a dual-mode transducer. Two raters outlined nerve margins on 110 paired B-Mode and SWE images every second for 20 s before and during injection. Bias and limits of agreement were plotted on Bland-Altman plots. We hypothesized that the bias of nerve diameter would be <2.5% and that the percent limits of agreement would lie ±0.67% (2 SD) of the bias.

**Results:**

There was no difference in the bias (95% confidence interval (CI) limits of agreement) of nerve diameter measurement, 0.01 (−0.14 to 0.16) cm, *P* = 0.85, equivalent to a 1.4% (−56.6% to 59.5) % difference. The bias and limits of agreement were 0.03 (−0.08 to 0.15) cm^2^, *P* = 0.54 for cross-sectional nerve area; and 0.02 (−0.03 to 0.07), *P* = 0.45 for shape. Reliability (ICC) between raters was 0.96 (0.94–0.98) for B-Mode nerve area and 0.91 (0.83–0.95) for SWE nerve area.

**Conclusions:**

Nerve diameter measurement from B-Mode and SWE images fell within a priori measures of bias and limits of agreement.

## Introduction

Accurate interpretation of ultrasound images is essential in order to conduct safe regional nerve block.^1^ However, studies routinely demonstrate failure of trainee anaesthetists to analyse images correctly^2^ and thereupon fail to adequately perform essential steps or avoid errors.^3,4^

Regional anaesthetists may also experience difficulty^5^ in discriminating tissue boundaries due to patient age, vascular disease, infection, and obesity.^6^

New technologies such as shear wave elastography (SWE) and artificial intelligence (AI)^7,8^ can aid identification of nerves by superimposing colour maps.

SWE is as an imaging modality used to diagnose breast^9^ and prostate^10^ cancer. It is a clinically established, validated, operator independent, ultrasound-based imaging tool that measures tissue elasticity. The underlying physical principle is that longitudinal acoustic radiation force generates shear waves, which propagate perpendicularly in tissue to the ultrasound beam. Young’s elastic modulus is proportional to the shear wave velocity squared.

Shear wave images are automatically co-registered with standard B-Mode images to provide quantitative colour elastograms that overlay anatomy.^11^ SWE is increasingly used to diagnose musculoskeletal disease because it shows high accuracy for discrimination between normal and stiff tissue^12^ and a threefold contrast between neural and extraneural tissue.^13^

Artificial intelligence forms one of the four grand challenges of UK industrial strategy. The Topol report recommended the application of digital technologies to the Healthcare industry, including AI.^14^ Application of AI to regional anaesthesia offers the potential to improve the quality and interpretation, automation, storage and linkage of data and images.^15^ Two clinical systems have been developed that use machine learning in order provide real-time colour overlay of soft tissue on a separate screen and present block-specific schematic anatomical images.^8,16^ One (ScanNav Anatomy Peripheral Nerve Block, Intelligent Ultrasound, Cardiff, UK) has Medicines and Healthcare Regulatory Agency (MHRA) approval for clinical use, but validation of nerve dimensions versus the gold standard technology has not been undertaken.

The American Society of Regional Anaesthesia (ASRA) has recommended scientific assessment of the technical capabilities of ultrasound equipment and its operators; and comparison of ultrasound to other methods of nerve localization.^1^

As a first step, we felt it pertinent to assess agreement between B-Mode and SWE images. We hypothesized that nerve diameter, cross-sectional area and shape were similar, and that the results would define the bias and level of agreement^17,18^ required for future validation of artificial intelligence systems.

Therefore, our primary objective was to plot the bias and limits of agreement of nerve diameter for paired B-Mode ultrasound and SWE images in patients receiving ultrasound-guided nerve block.

## Methods

### Ethics approval

Eleven patients underwent ultrasound-guided nerve blocks for elective orthopaedic limb surgery at Ninewells Hospital and University of Dundee School of Medicine. The East of Scotland Medical Ethics committee stated in writing that no formal ethical approval process needed to be undertaken because data were collected as part of normal practice and did not interfere with routine clinical care. We obtained authority from our local Caldicott Guardian to collect our elastography images (Data Protection Reg No Z8537226). In the United Kingdom, Caldicott Guardians are appointed to take responsibility for data governance and ensure patient confidentiality for all forms of data collection, particularly for studies not requiring full ethical approval but intending to be published. Study registration was not sought because we used paired data and did not randomize to different groups.

Nerve block was performed using a linear SWE 5 MHz to 12 MHz ultrasound transducer by 11 anaesthetists. All were consultant anaesthetists competent in use of ultrasound for regional nerve block. The Aixplorer ultrasound machine (Supersonic Imagine, Aix-en-Provence, France) generated a B-Mode and an SWE image. The anaesthetists only used the B-Mode image for nerve recognition and needle alignment. None had knowledge or experience of SWE.

The following nerve blocks were performed: axillary radial (*n* = 3), femoral (*n* = 3), infraclavicular (*n* = 2), interscalene (*n* = 1), sciatic (*n* = 1) and supraclavicular (*n* = 1). Injection technique, choice of local anaesthetic concentration and volume were at the discretion of each anaesthetist. The rate of injection was approximately 15 mL.min^-1^. No adjuvants were given. Routine medication such as analgesics, anti-emetics and anti-hyperalgesics were administered according to local guidelines.

### Measurement of nerve area

We recorded cine loops of the paired B-Mode and SWE images immediately before and during injection of 5 mL bolus injection of local anaesthetic over 20 s. Procedures were recorded onto the hard drive of the ultrasound machine and stored as Digital Imaging and Communications in Medicine (DICOM) videos. For analysis, videos were converted to individual Tagged Image File Format (TIFF) frames using Adobe Premier Pro video editing software (Adobe, CA). Each image was standardized for depth at 8.4 pixels cm^-1^. Using the freehand drawing tool of ImageJ (v1.47, NLM, Washington), the cross-sectional nerve area of each nerve was measured 20 times at 1-s intervals on B-Mode and SWE videos by a single rater. Image J automatically calculated the diameter and shape of the nerve. Shape was defined as ‘roundness’ and calculated by the equation 4 × area/(π × major axis)^2^, where the major axis was the primary axis of a best fitting ellipse. This calculation equates to minor axis/major axis – the inverse of the aspect ratio.

The principal rater was a medical student on a vocation scholarship investigating the application of SWE to regional anaesthesia. She was trained in B-Mode and SWE ultrasound by the senior investigator, an experienced regional anaesthetist before starting the study.

### Reliability of measurement

A second rater, a consultant regional anaesthetist, using the freehand tool of ImageJ, measured cross-sectional nerve areas every 4 s during injection.

Therefore, our secondary objectives were to measure cross-sectional nerve area and shape, correlation between B-Mode and SWE measures and change in nerve dimensions over time and demonstrate contrast between epineural and perineural tissue using B-Mode imaging and SWE.

### Statistical analysis

From previous research using B-Mode ultrasound and SWE on soft embalmed Thiel cadavers,^19,20^ we defined bias as 2.5% and 95% levels of agreement < 67% of the bias. The latter represented 2 SD of the bias. In order to test agreement between repeated measurements using B-Mode and SWE, we calculated residual (within) subjects standard deviation using a repeated measures, mixed models analysis (Number Cruncher Statistical System v10, NCSS, Utah) and lme4 package of R, according to the recommendations of Myles.^17^ Mode of ultrasound (B-Mode or SWE) was a fixed effect; and nerve as a random effect. We plotted (GraphPad Prism 5, GraphPad, CA) mean B-Mode and SWE paired values of nerve diameter, area and shape on the *x-axis* and their differences on the *y-axis*. The Bland-Altman bias and 95% limits of agreement were presented graphically. Inter-observer reliability was measured using intraclass correlation, calculated using a two-way model for rater agreement in the irr package of R. Spearman’s test was used for correlation of paired data.

### Power analysis

We made no presumptions regarding the nature and distribution of the data. We thought that paired B-Mode and SWE data measured repeatedly 20 times on each patient would provide us with sufficient data for presentation of Bland-Altman graphs. Therefore, we analysed 11 patients in order to account for any technical problems.

## Results

### General observations

**Figure 1.**
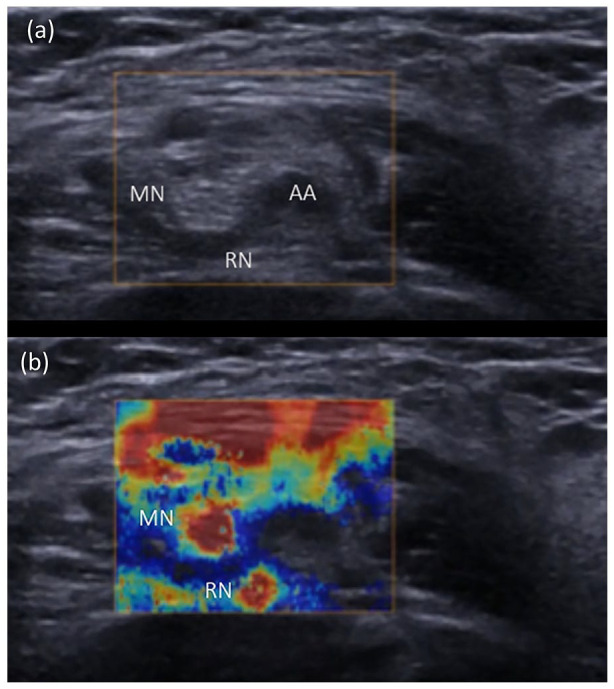
Axillary SWE (above) and B-Mode (below) images. The SWE image illustrates two red, circular areas at 9 o’clock and at 7 o’clock to the axillary artery (AA), consistent with the positions of the median nerve (MN) and radial nerves (RN), respectively in the B-Mode image below. Some superficial patterns were generated secondary to tissue strain.

Injection of local anaesthetic was uneventful. All local anaesthetic spread around nerves and was visualized clearly without air bubbles. No intraneural injection occurred. Paired B-Mode and SWE images from the axillary brachial plexus are shown in Figure 1(a) and (b). The median and radial nerves are seen as a distinct, red, round or elliptical structure surrounded by a thin yellow/green halo.

### Nerve dimensions

**Figure 2.**
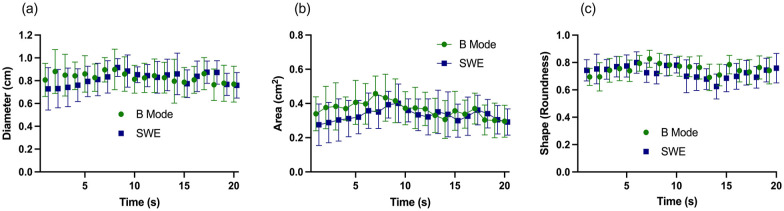
Nerve diameter, area and shape measured using B-Mode ultrasound and SWE. Mean (95% CI).

In total, 440 measurements of cross-sectional nerve area were made, divided equally between B-Mode and SWE and two raters. There was no difference, respectively, in mean (95% confidence interval (CI)) nerve diameter, difference 0.01 (95% CI: −0.14 to 0.16), *P* = 0.85; cross-sectional nerve area, difference 0.03 (95% CI: −0.08 to 0.15), *P* = 0.54; or shape, difference 0.02 (95% CI: −0.03 to 0.07), *P* = 0.45 (Table 1). Nerve diameter and cross-sectional area were similar in 9 out of 11 nerves, and shape similar in 8 out of 11 nerves. There was no difference in nerve diameter, area or shape over time (Figure 2).

**Table 1.** Nerve dimensions. Mean (95% CI) nerve diameter, cross-sectional nerve area, and shape from 11 patients. Mean and 95% CI calculated from 20 repeated measurements over 20 s at 1-s intervals. Shape represents roundness calculated as: 4 × area/(π × major axis)^2^, where the major axis is the primary axis of a best fitting ellipse.

	Feret diameter (cm)			Cross-sectional area (cm^2^)			Shape (roundness)		*P*
	B-Mode (cm)	SWE (cm)	*P*	B-Mode (cm^2^)	SWE (cm^2^)	*P*	B-Mode	SWE	
All nerves	0.83 (0.80–0.86)	0.82 (0.79–0.84)	0.85	0.36 (0.34–0.39)	0.33 (0.31–0.35)	0.54	0.75 (0.73–0.77)	0.73 (0.71–0.75)	0.45
Femoral 1	0.85 (0.75–0.91)	0.84 (0.77–0.91)	0.72	0.42 (0.36–0.43)	0.38 (0.33–40.3)	0.11	0.80 (0.76–80.4)	0.76 (0.70–0.20)	0.24
Femoral 2	0.63 (0.60–0.66)	0.66 (0.59–0.72)	0.40	0.23 (0.20–0.26)	0.21 (0.19–0.24)	0.20	0.80 (0.76–0.84)	0.75 (0.68–0.81)	0.12
Femoral 3	0.98 (0.92–1.05)	0.92 (0.85–0.99)	0.14	0.51 (0.46–0.56)	0.42 (0.37–0.49)	0.01	0.76 (0.70–0.81)	0.78 (0.73–0.84)	0.34
Lateral infraclavicular 1	0.63 (0.59–0.67)	0.73 (0.65–0.81)	0.04	0.22 (0.19–0.25)	0.21 (0.17–0.24)	0.69	0.79 (0.73–0.85)	0.69 (0.62–0.76)	0.004
Lateral infraclavicular 2	0.61 (0.58–0.63)	0.69 (0.60–0.77)	0.08	0.22 (0.19–0.25)	0.19 (0.15 –0.22)	0.11	0.83 (0.79–0.86)	0.68 (0.61–0.75)	0.0003
Interscalene C6	0.71 (0.64–0.78)	0.66 (0.60–0.73)	0.26	0.27 (0.22–0.31)	0.25 (0.21–0.29)	0.44	0.74 (0.69–0.78)	0.79 (0.73–0.84)	0.21
Axillary radial 1	0.84 (0.77–0.91)	0.80 (0.74–0.86)	0.24	0.36 (0.31–0.42)	0.28 (0.23–0.33)	0.002	0.78 (0.72–0.83)	0.68 (0.61–0.74)	0.03
Axillary radial 2	0.73 (0.68–0.77)	0.69 (0.63–0.74)	0.22	0.29 (0.26–0.32)	0.25 (0.21–0.29)	0.07	0.80 (0.76–0.84)	0.76 (0.71–0.82)	0.28
Axillary radial 3	0.95 (0.90 –1.00)	0.96 (0.90–1.04)	0.69	0.45 (0.39–0.51)	0.46 (0.40–0.51)	0.78	0.69 (0.63–0.74)	0.69 (0.64–0.74)	0.98
Sciatic	1.19 (1.12 –1.26)	1.03 (0.99–1.08)	0.005	0.59 (0.53–0.65)	0.54 (0.49–0.60)	0.29	0.59 (0.52–0.65)	0.75 (0.69–0.81)	0.0002
Supraclavicular	1.00 (0.93–1.07)	1.00 (0.94–1.06)	0.99	0.47 (0.40–0.54)	0.46 (0.41–0.51)	0.78	0.68 (0.63–0.73)	0.71 (0.66–0.76)	0.48

Inter-observer reliability (ICC) between raters was 0.96 (0.94–0.98) for B-Mode nerve area and 0.91 (0.83–0.95) for SWE nerve area.

**Figure 3.**
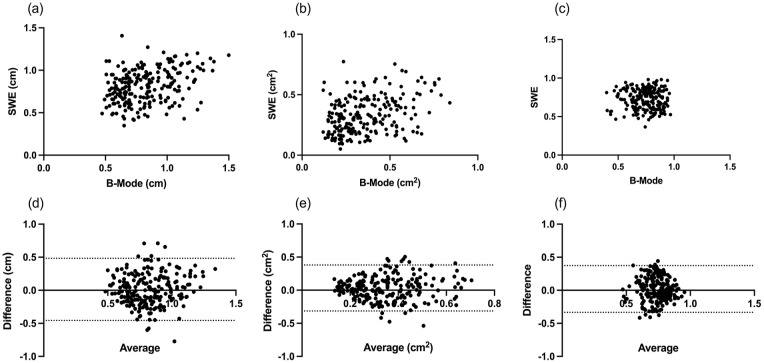
Nerve dimensions – correlation and Bland-Altman plots. Scatter plots of paired nerve dimensions calculated from B-Mode and SWE images. Images (a) to (c) show correlation between B-Mode and SWE measurements of nerve diameter (a), cross-sectional nerve area (b), and nerve shape or roundness (c). Images (d) to (f) show Bland-Altman plots of nerve diameter (d), cross-sectional nerve area (e), and nerve shape or roundness (f). The bias (95% limits of agreement) were 0.01 (−0.46 to 0.48); 0.03 (−0.31 to 0.38) and 0.02 (–0.33 to 0.37), respectively.

Correlations (ρ) between B-Mode and SWE measures of diameter, area and shape were 0.31 (0.18–0.43), *P* < 0.0001; 0.32 (0.20–0.44), *P* < 0.0001, respectively. There was no correlation between measures of shape 0.02 (−0.16 to 0.11), *P* = 0.69 (Figure 3).

Scatter plots showing bias and levels of agreement for diameter, cross-sectional nerve area and shape are shown in Figure 3. Scatter was random and did not display any trend. Bias (95% levels of agreement) were 0.01 (−0.46 to 0.48); 0.03 (−0.31 to 0.38) and 0.02 (–0.33 to 0.37) for diameter, area and shape, respectively.

**Figure 4.**
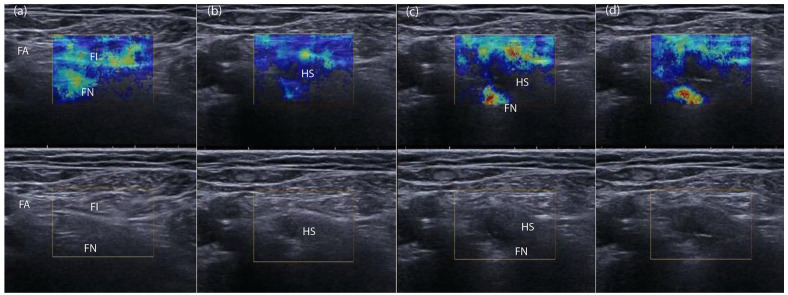
Local anaesthetic injection captured on paired B-Mode and SWE images during femoral block. The relatively stiff femoral nerve (FN) and fascia iliaca (FI) are seen in colour. The femoral nerve was difficult to see using B-Mode ultrasound, but, using SWE, was identified underneath the distinct coloured sweep of the fascia iliaca, lateral to the femoral artery (a). Initial injection (b) of local anaesthetic was associated with separation of the femoral nerve and fascia iliaca and reduction in colour intensity over the fascia iliaca and femoral nerve, albeit the epineurium remained discernible as a light blue colour with increased intensity inferiorly. With further injection, the anechoic hydrolocation space (HS) created by local anaesthetic injection is visible between the fascia iliaca and femoral nerve (c), and nerve stiffness increases in image (d) as local anaesthetic compresses the femoral nerve against the ilio-psoas muscle.

Figure 4 illustrates the dynamic changes in SWE during local anaesthetic injection. The femoral nerve was difficult to see using B-Mode ultrasound, but, using SWE, was identified underneath the distinct coloured sweep of the fascia iliaca, lateral to the femoral artery. Injection of 5 mL local anaesthetic was associated with separation of the femoral nerve and fascia iliaca and reduction in colour intensity over the fascia iliaca and femoral nerve, albeit the epineurium remained discernible as a light blue colour with increased intensity inferiorly.

### Three-dimensional representation

**Figure 5.**
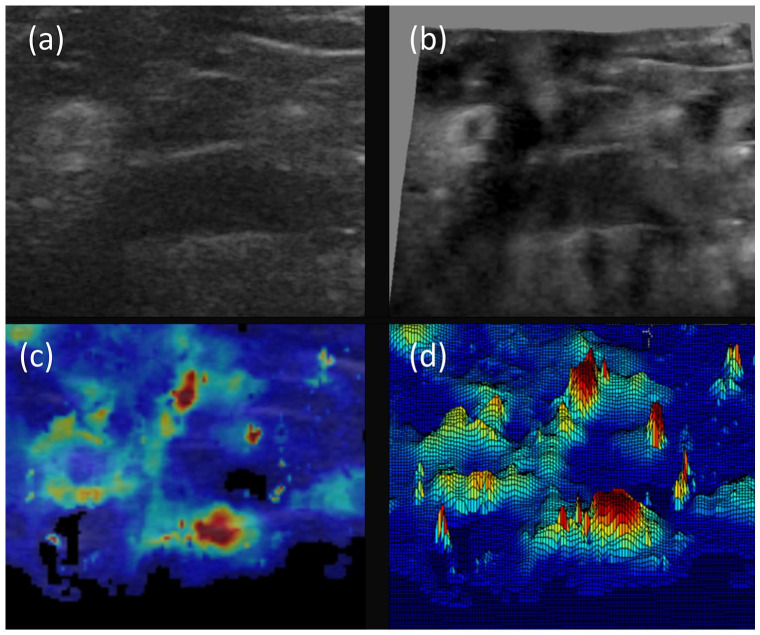
Relative contrast of B-Mode and SWE images using 3D representation of images taken from infraclavicular block. Image (a) shows B-Mode ultrasound image and image (b) shows 3D representation using ImageJ with brightness (0–255) on the *z-axis*. Image (c) shows SWE image and image (d) shows 3D SWE representation using research software. Note the marked improvement in contrast using SWE and distinct stiff epineural borders of lateral and posterior cords. Also note the reduction in nerve stiffness within the centre of the lateral cord.

Three-dimensional (3D) representation of B-Mode and SWE images (Figure 5) highlights the relative contrast using both imaging modalities. B-Mode images show small contrast because formation of a B-Mode ultrasound image is dependent on acoustic impedance. The 3D representation of the SWE image offers a detailed map of elasticity and greater contrast between epineurium and subepineural and perineural tissue.

## Discussion

Limits of agreement of nerve dimensions using B-Mode ultrasound and SWE fell within the bias of <2%, and >95% of data fell within our 67% limits of agreement for nerve diameter. We present a standard for anatomical comparison using future technologies, including AI, using Bland-Altman analysis.^17,18^

The strength of our study was that we sought to define criteria for future technological development by using SWE, an advanced ultrasound technology that measures elasticity^11^ as well as the brightness of tissue interfaces, and offers informative 3D representations of tissue. Elasticity was used as a measure of nerve dimensions because it is a validated, operator independent tool^11^ used to accurately diagnose stiff tumours such as breast and prostate cancer, and increasingly is used to investigate soft tissue disorders.

Moreover, SWE, on the other hand, does not deteriorate with patient age, illness or obesity because images are formed from measures of tissue elasticity, a fundamental physical property of tissue.^21^ SWE differentiates stiff epineurium threefold from soft perineural tissue,^13^ and thus provides a colour border that can be traced and measured.

We defined our limits of agreement from previous cadaver studies^19^ and assumed that the true diameter, cross-sectional area and shape of the nerve were not known, and that neither B-Mode imaging or SWE represented the gold standard measurement modality. We also acknowledged that our measurements could be subject to error. Nevertheless, we are reassured by the high intraclass correlations between raters. ImageJ has an automatic measurement facility but requires conversion of the image to a binary black and white image using a grayscale threshold. We attempted this initially but found that the threshold had to be constantly altered in order to capture reliable images.

The product–moment correlation coefficient (*r*) between the results of two measurement methods is sometimes used as an indicator of agreement. However, correlation measures the association between two variables, and not the comparability between methods. Similarly, *r*^2^, named the coefficient of determination, only gives the proportion of variance that two variables have in common.

Performance of the block was undertaken by 11 consultant anaesthetists experienced in ultrasound-guided nerve block who used the B-Mode image for guidance. The SWE image was hidden, and thus any differences between modalities in delineation of hydrolocation or needle tip position were not relevant.

The advantage of SWE was that nerves that can be difficult to recognize or outline in practice become recognizable because nerve epineurium is 3 times stiffer than surrounding connective tissue.^13^ In fact, our SWE was so sensitive, it showed a reduction in stiffness within the subepineurium of the lateral cord (Figure 4). Maps of Young’s modulus revealed marked differences in tissue stiffness at the nerve border, more so distally at the infraclavicular posterior cord, axillary radial and femoral nerves. Proximally in the brachial plexus the opposite was seen; stiffer connective tissue was identified circumferentially around the nerve roots and trunks but with limited colour identification within these structures.

Colour maps had sufficient resolution to identify epineurium but not intraneural structures and thus limit the application of this technology to ultrasound guidedc regional anaesthesia (UGRA) in the meantime.

Application of shear wave elastography to UGRA had some practical imitations. The probe is bulkier than standard B-Mode ultrasound probe and requires a very light touch in order to minimize artefact. Superficial colour changes are probably indicative of external compression but unsurprising given the lack of experience of operators using this mode of ultrasound. We think it unlikely that axial strain contributed to any error in measurement as Young’s Elastic Modulus is derived from a threefold multiplication of transverse Shear Modulus, the product of tissue density and the square of transverse wave velocity.

We recognize that current AI systems for regional anaesthesia are within their infancy. Artificial intelligence research so far has focused on binary recognition or not of the nerve, and anaesthetist recognition of anatomy from static images. Authors have hypothesized that anatomical recognition could be used as a means of assessment and be translated as an aid in clinical practice.

Machine learning (ML), a subset of artificial intelligence (AI), has been proposed as a tool to aid novice anaesthetists recognize anatomical structures, reduce procedural cognitive load, improve learning, and ultimately augment clinical practice.^7,22^ Several studies have used convolutional neural networks^15,23,24^ in order to detect the spatial and temporal features of the median and sciatic nerves on images obtained from patients and soft embalmed cadavers, and claimed 80% to 94% successful identification of nerves. Two advanced systems^7,22^ have been developed using ML that present nerves, arteries, muscle and fascia in different colours, but the accuracy and reliability of images nor agreement with other modalities has not been investigated.

However, for any technology to have an impact, it must improve on the current standard. New technology should, for instance, either enhance block efficacy, accelerate learning curves or reduce side effects. Moreover, one would expect a new imaging technology would not just simply recognize gross anatomy, but improve the contrast between epineurium and adjacent structures such as fascia, veins and arteries, in order to reduce the accidental epineural needle contact and subperineural local anaesthetic injection. Therefore, accurate recognition of epineurium and fascial planes and confirmation of injection site is essential, otherwise any learning or translation to clinical practice will be erroneous and potentially cause patient harm.

This may be difficult to achieve using a 256 grayscale. B-Mode images offer a relatively poor level of tissue contrast, technically termed acoustic impedance, that is likely to diminish in the old, frail, ill and obese patients most in need of nerve block. Thus, it demonstrates a fundamental flaw in the application of AI to regional anaesthesia and ultrasound imaging in general. Use of physical properties such as stiffness has the potential to not only improve subjective recognition of nerves but also improve AI recognition of nerves.

We acknowledge the role of AI in teaching basic, introductory ultrasound anatomy teaching, but recommend that it should be developed as a means of recognizing deep seated structures in obese individuals.

Moreover, we foresee AI not as a sole technology, but as one of many interventions that can augment skills such as touch, needle feedback and resistance to injection in order to identify the correct needle tip position for injection.

## Conclusion

SWE is an accurate and reliable tool for measurement of cross-sectional nerve area, diameter and shape. We present acceptable bias and limits of agreement using SWE that future AI should fulfil. This way we can ensure that clinical application of AI to interpretation of nerves and soft tissues is set to the highest standard, and can augment clinical decision making, thus improving both efficacy of intervention and reducing side effects.

## References

[1] Neal JM Brull R Horn JL , et al. The second American society of regional anesthesia and pain medicine evidence-based medicine assessment of ultrasound-guided regional anesthesia: executive summary. Reg Anesth Pain Med 2016; 41: 181–194. 26695878 10.1097/AAP.0000000000000331

[2] Liu SS YaDeau JT Shaw PM , et al. Incidence of unintentional intraneural injection and postoperative neurological complications with ultrasound-guided interscalene and supraclavicular nerve blocks. Anaesthesia 2011; 66: 168–174. 21320084 10.1111/j.1365-2044.2011.06619.x

[3] Chuan A Graham PL Wong DM , et al. Design and validation of the regional anaesthesia procedural skills assessment tool. Anaesthesia 2015; 70: 1401–1411. 26558857 10.1111/anae.13266

[4] McLeod GA McKendrick M Taylor A , et al. An initial evaluation of the effect of a novel regional block needle with tip-tracking technology on the novice performance of cadaveric ultrasound-guided sciatic nerve block. Anaesthesia 2020; 75: 80–88. 31506921 10.1111/anae.14851

[5] McKendrick M Sadler A Taylor A , et al. The effect of an ultrasound-activated needle tip tracker needle on the performance of sciatic nerve block on a soft embalmed Thiel Cadaver. Anaesthesia 2021; 76: 209–217. 32797700 10.1111/anae.15211

[6] Hoskins P Martin K Thrush A , et al. Diagnostic ultrasound, third edition: physics and equipment. Boca Raton, FL: CRC Press, 2019.

[7] Bowness J El-Boghdadly K Burckett-St Laurent D . Artificial intelligence for image interpretation in ultrasound-guided regional anaesthesia. Anaesthesia 2021; 76: 602–607. 32726498 10.1111/anae.15212

[8] Gunaydin B Gungor I Inan G . Accuracy study design: assistive AI, ultrasound-guided block. J Anesth 2021; 35: 603. 34213629 10.1007/s00540-021-02966-0

[9] Evans A Sim YT Pourreyron C , et al. Pre-operative stromal stiffness measured by shear wave elastography is independently associated with breast cancer-specific survival. Breast Cancer Res Treat 2018; 171: 383–389. 29858751 10.1007/s10549-018-4836-5 PMC6096877

[10] Ageeli W Wei C Zhang X , et al. Quantitative ultrasound shear wave elastography (USWE)-measured tissue stiffness correlates with PIRADS scoring of MRI and Gleason score on whole-mount histopathology of prostate cancer: implications for ultrasound image-guided targeting approach. Insights Imaging 2021; 12: 96. 34236553 10.1186/s13244-021-01039-w PMC8266979

[11] Cosgrove D Piscaglia F Bamber J , et al. EFSUMB guidelines and recommendations on the clinical use of ultrasound elastography. Part 2: clinical applications. Ultraschall Med 2013; 34: 238–253. 23605169 10.1055/s-0033-1335375

[12] Alfuraih AM O’Connor P Tan AL , et al. Muscle shear wave elastography in idiopathic inflammatory myopathies: a case-control study with MRI correlation. Skeletal Radiol 2019; 48: 1209–1219. 30810778 10.1007/s00256-019-03175-3 PMC6584706

[13] Munirama S Eisma R Columb M , et al. Physical properties and functional alignment of soft-embalmed Thiel human cadaver when used as a simulator for ultrasound-guided regional anaesthesia. Br J Anaesth 2016; 116: 699–707. 27106974 10.1093/bja/aev548

[14] Topol E . The Topol review: preparing the healthcare workforce to deliver the digital future, 2019, https://topol.hee.nhs.uk/

[15] McKendrick M Yang S McLeod GA . The use of artificial intelligence and robotics in regional anaesthesia. Anaesthesia 2021; 76(Suppl. 1): 171–181. 33426667 10.1111/anae.15274

[16] Bowness J Varsou O Turbitt L , et al. Identifying anatomical structures on ultrasound: assistive artificial intelligence in ultrasound-guided regional anesthesia. Clin Anat 2021; 34: 802–809. 33904628 10.1002/ca.23742

[17] Myles PS Cui J . Using the Bland-Altman method to measure agreement with repeated measures. Br J Anaesth 2007; 99: 309–311. 17702826 10.1093/bja/aem214

[18] Carstensen B Simpson J Gurrin LC . Statistical models for assessing agreement in method comparison studies with replicate measurements. Int J Biostat 2008; 4: 16. 22462118 10.2202/1557-4679.1107

[19] Joy J McLeod G Lee N , et al. Quantitative assessment of Thiel soft-embalmed human cadavers using shear wave elastography. Ann Anat 2015; 202: 52–56. 26342463 10.1016/j.aanat.2015.06.007

[20] McLeod G McKendrick M Taylor A , et al. Validity and reliability of metrics for translation of regional anaesthesia performance from cadavers to patients. Br J Anaesth 2019; 123: 368–377. 31255289 10.1016/j.bja.2019.04.060

[21] Wells PN Liang HD . Medical ultrasound: imaging of soft tissue strain and elasticity. J R Soc Interface 2011; 8: 1521–1549. 21680780 10.1098/rsif.2011.0054 PMC3177611

[22] Gungor I Gunaydin B Oktar SO , et al. A real-time anatomy identification via tool based on artificial intelligence for ultrasound-guided peripheral nerve block procedures: an accuracy study. J Anesth 2021; 35: 591–594. 34008072 10.1007/s00540-021-02947-3 PMC8131172

[23] Alkhatib M Hafiane A Tahri O , et al. Adaptive median binary patterns for fully automatic nerves tracking in ultrasound images. Comput Methods Programs Biomed 2018; 160: 129–140. 29728240 10.1016/j.cmpb.2018.03.013

[24] Alkhatib M Hafiane A Vieyres P , et al. Deep visual nerve tracking in ultrasound images. Comput Med Imaging Graph 2019; 76: 101639. 31349184 10.1016/j.compmedimag.2019.05.007

